# Introduction to the Special Issue on Cascading Disaster Modelling and Prevention

**DOI:** 10.3390/ijerph18094813

**Published:** 2021-04-30

**Authors:** Thomas J. Huggins, Lili Yang, Didier Sornette

**Affiliations:** 1 Division of Science & Technology, BNU-HKBU United International College, Zhuhai 519087, China; tjhuggins@uic.edu.cn; 2 Academy for Advanced Interdisciplinary Studies, Southern University of Science and Technology, Shenzhen 518055, China; 3 Department of Statistics and Data Science, Southern University of Science and Technology, Shenzhen 518055, China; 4 Department of Finance, Southern University of Science and Technology, Shenzhen 518055, China; dsornette@ethz.ch; 5 Department of Management, Technology and Economics, ETH Zurich, CH-8092 Zurich, Switzerland

The 2019 Global Assessment Report (GAR2019) on Disaster Risk Reduction [1] defined cascading hazard processes as, “a primary impact (trigger) such as heavy rainfall, seismic activity or unexpectedly rapid snow melt, followed by a chain of consequences that can cause secondary impacts” (p. 49). Together with the antecedent 2015 Sendai Framework for Disaster Risk Reduction [2], the GAR2019 promoted a focus on linkages between hazard types, where effective disaster management helps ensure that initial events do not lead to more challenging response and recovery scenarios. This is achieved by mitigating the links, for example, between flooding and roading infrastructure failure so that response vehicles can still navigate an affected area, and so that evacuation remains viable at several stages. As exemplified by Santella et al. [3], the identification and prioritization of certain linkages remains paramount. However, as stated by Pescaroli and Alexander [4], “critical infrastructure and complex adaptive systems may amplify the impacts of the cascade” (p. 2250). This adds many other layers of complex interactions to mean that even the most experienced and well-trained disaster managers cannot make effective decisions without analytical support.

The current special issue primarily focuses on how cascading disaster risk can be analyzed and modeled—to support decision-making about disaster mitigation, preparation, and response. The special issue covers cascading impacts triggered by multiple hazard types, cascading impacts triggered by extreme rainfall events and by ground deformation, and the cascading impacts of business and infrastructure disruptions. Specific issues concerning the risk of cascading hazards for oil storage infrastructure and for underground railways are also analyzed, alongside potential mitigation measures. Other papers outline the importance of organization within social networks and of public health provision for cascading disaster management.

Mignan and Wang [5] analyzed the occurrence of 24 different types of natural, socioeconomic, and health-related hazard events, in a set of 29 cascading disaster events. Their analysis accounted for both unidirectional triggers and feedback loops between one hazard event and another. Results identified how network failure and business interruption have become key components in the propagation of cascading disaster impacts. The same analysis demonstrated how historical hazard events have triggered a broad range of natural, technological, and socioeconomic impacts. Analytical challenges remain for estimating the timeframe of event-to-event interactions and for addressing multifactorial dynamics [5], for example: the dual effects of wind and drought on wildfires.

Huggins et al. [6] focused on cascading disaster propagation from one type of initial trigger: extreme rainfall. They focused on this trigger due to the increasing prevalence of storm surge and other cascading events impacting coastal cities around the world. PRISMA [7] guidelines were employed to conduct a systematic literature review of 99 relevant case studies, published in peer-reviewed academic literature. Twenty-five different kinds of linkages were identified, ranging between natural hazard triggers and infrastructural impacts. To support more comprehensive analyses of rain-related cascading dynamics, the authors have generated a matrix of potential trigger-impact relationships that can be populated with localized data or expert opinion ratings. The resulting data could then be used for a range of analyses, including the network analysis employed by Mignan and Wang [5], and both multi-level [8] and multi-system [9] analyses.

Cando-Jácome et al. [10] used an earthquake in Ecuador, in April 2015, to demonstrate how earthquakes interact with severe land deformation and can lead to the collapse of built infrastructure. In this case, seismically induced deformation led to the collapse of a large shopping center, causing at least 96 deaths. The authors demonstrated how the risk of this kind of cascading disaster is determined through synthetic aperture radar interferometry (InSAR), which uses differences between two satellite images to establish degrees of deformation. The authors combined this type of spatial analysis with an analysis of population flow, transit, displacement, accessibility, and concentration to generate a map of building collapse risk. This technique was able to identify areas of Quito City where the population faces a high and complex risk of building collapse. The authors’ use of generally available data and replicable analysis means that the same method may help map the risk of building collapse in many other cities around the world.

Dubaniowski and Heinimann [9] used an elaborate network analysis to more closely analyze the type of business and infrastructure disruptions highlighted by Mignan and Wang [5]. A set of simulations was used to establish an overall system-of-systems model for a range of hazard events. The authors addressed a concern raised by Mignan and Wang [5] by determining that 113% of expected recovery time was the optimal granularity for simulating interactions between relevant nodes and systems. These timeframes allowed Dubianowski and Heinimann [9] to account for the propagation of diverse inter-system impacts between business and infrastructural networks.

Zhang et al. [11] developed a full-scale, three-dimensional model of a large oil depot site in Shenzhen, China. They used a combination of numerical methods to simulate the progression, starting with heavy rainfall, and then followed by landslide, pipeline breakage, oil leak, and explosion. This analysis showed that even one cubic meter of landslide mass could lead to a naTech disaster, hallmarked by a highly destructive explosion. The same simulation was able to identify the spatial distribution of impacts and gauge thresholds for each trigger in the chain of hazard events. This approach to simulation provided a vivid demonstration of infrastructure vulnerability and key points for cascading disaster mitigation.

Wang et al. [12] analyzed another specific context for cascading disasters: the underground railway system in Beijing, China. Frequent passenger-related incidents have created significant and often complex disruptions to large-scale transportation. Like other papers published in the current special issue, the challenge was to identify the most critical hazards in question. This was achieved by breaking down a set of 327 accident case studies into interactions between specific passenger tasks. A task-orientated accident investigation process and complex network analysis was used to identify several hazardous behaviors and other factors that were clearly mitigable as a part of railway hazard management. The resulting analysis can be updated with even more recent accident data to ensure that metro safety managers can dynamically prioritize and manage critical hazards.

Chen et al. [13] focused on the management of a single disaster event, which had the potential to become a cascading disaster. They applied social network analysis to data from the April 2013 Ya’an earthquake in China. The authors state that disaster management capacities had been strengthened following the catastrophic 2008 Wenchuan Earthquake, which had triggered catastrophic impacts in the same province. New capacities included the ability to coordinate between 122 organizations working to respond to, and recover from, the 2013 earthquake. Network analysis identified that the Sichuan state-level government had the highest level of degree centrality and between centrality of any other network node (0.846 and 0.087, respectively). For Chen et al. [13], this marks the key role played by the state government, and the support provided by a wide range of other organizations.

Harada et al. [14] provided a case study of support required following the 2011 Great East Japan Earthquake and Tsunami. They gathered qualitative data from 435 of 1911 dietitians who supported the affected population during the aftermath. These participants completed an open-ended questionnaire that asked them to “Please write freely about the support that you wanted at the time of the disaster.” Qualitative descriptive analysis was then used to extract four themes, concerning: physical resources, pre-established system, information, and human resources. Participants most frequently required the physical resources of “food” (65 instances) and “gasoline” (59 instances). They reported that these resources, alongside human resources and information, were difficult to acquire due to the lack of pre-established systems. On one hand, these findings highlight a capacity gap that characterizes all major disasters—as a serious disruption in functioning, exceeding the capacity of communities to respond [15]. On the other hand, Harada et al. [14] highlight the need for more flexible and upwardly scalable disaster management, which can better meet the shifting challenges of a major, cascading disaster event.

The current special issue on Cascading Disaster Modelling and Prevention includes several examples of how cascading disaster analysis supports a more preventative and pro-active approach to cascading disaster risk. Case study reviews and other data analyses have illustrated how cascading impacts are triggered by multiple hazard types, and can be characterized by severe infrastructural and organizational disruptions. Relevant analyses have been conducted for specific sites, namely oil depots and underground railway systems. In both cases, innovative approaches to analytical modeling have identified key targets for cascading hazard mitigation. The remaining pair of special issue papers highlight the potential to learn from prior disasters, which have already affected large populations and broad geographical areas. Flexible and well-networked capacities remain paramount, especially where mitigation has not been achieved or where key targets for mitigation have not yet been identified. Analytical efforts are ongoing to help ensure that larger scale cascading disaster management can leverage more of the analytical precision that characterizes site-specific analyses.
